# Effects of Dysesthesia-Matched Transcutaneous Electrical Nerve Stimulation on Postural Control After Lumbar Spinal Stenosis Surgery: A Case Report

**DOI:** 10.7759/cureus.88186

**Published:** 2025-07-17

**Authors:** Katsuma Shoka, Shogo Sawamura, Koki Ikuno

**Affiliations:** 1 Department of Rehabilitation/Physical Therapy, Central Japan International Medical Center, Minokamo, JPN; 2 Department of Rehabilitation, Heisei College of Health Sciences, Gifu, JPN; 3 Department of Rehabilitation, Nishiyamato Rehabilitation Hospital, Kawai, JPN

**Keywords:** dysesthesia, lumbar spinal stenosis, plantar dysesthesia, postural control, stabilometry, transcutaneous electrical nerve stimulation

## Abstract

Persistent plantar dysesthesia after lumbar spinal stenosis (LSS) surgery impairs balance and increases fall risk. A 70-year-old woman with bilateral plantar dysesthesia (numerical rating scale (NRS) 8/10) received dysesthesia-matched transcutaneous electrical nerve stimulation (DM-TENS; 80 Hz, 100 µs) twice daily from postoperative day (POD) 15 to 30 (15 sessions) in conjunction with standard rehabilitation. Clinical assessments (e.g., muscle strength, ambulation, and activities of daily living (ADLs)) were performed on POD 2, 14, and 30. Psychological and postural control assessments were conducted on POD 14 and 30. Dysesthesia improved (NRS 8→3), ADL performance (Canadian occupational performance measure) increased (3→8), psychological distress decreased (PCS 25→16; HADS 21→18), and functional ambulation category improved (3→5). Under eyes-closed conditions, sway-ellipse area, path length, and mean velocity decreased by 24.2%, 25.9%, and 25.8%, respectively. DM-TENS was associated with rapid relief of dysesthesia and objective improvements in postural control, suggesting its utility as an adjunctive therapy for persistent post-LSS dysesthesia and balance impairment.

## Introduction

Lumbar spinal stenosis (LSS) primarily arises from age-related degenerative spinal changes. Its prevalence is increasing, particularly in aging populations, such as those in Japan [1]. LSS substantially affects patients’ activities of daily living (ADL) and quality of life (QOL) and requires effective management. Neurogenic intermittent claudication, a hallmark of LSS, often involves lower extremity pain/dysesthesia during activity that is relieved by rest. Severe LSS can cause cauda equina syndrome, presenting with bladder/bowel dysfunction, lower-extremity weakness, and bilateral plantar dysesthesia, which often persists postoperatively [2]. Patients experiencing dysesthesia, even at rest, exhibit greater ADL/QOL decrements than those without [3]. Chronic dysesthesia can increase sensory abnormalities and discomfort, thereby increasing the risk of stress, anxiety, and depression [4]. Managing LSS-related dysesthesia pre- and postoperatively is challenging.

Current management strategies include neuropathic pain medications such as pregabalin and duloxetine, although their efficacy for LSS remains uncertain [5]. Gabapentin, in contrast, has shown moderate efficacy in randomized trials [5]. Physical modalities, including electrical stimulation and thermotherapy, as well as physical therapy (PT) strategies such as flexion-based exercises, aquatic therapy, and nerve mobilization, have been shown to benefit patients by improving walking capacity and reducing symptom severity [5]. Although decompressive surgery is effective for relieving mechanical compression, sensory deficits such as plantar dysesthesia may persist postoperatively. In a large cohort, approximately 10-20% of patients reported worsening symptoms after three years of conservative follow-up, and little is known about the full extent of sensory recovery even after surgery [5]. These findings underscore the need for adjunctive rehabilitation strategies to address residual sensory impairment. Our clinical experience also suggests that persistent dysesthesia often undermines motivation and delays functional recovery, even when decompression is anatomically successful. However, persistent plantar dysesthesia can degrade the proprioceptive input, which is associated with impaired postural control, reduced gait, and increased fall risk [6]. The reduced proprioceptive input from peripheral neuropathy may increase spontaneous postural sway and visual dependency on balance [6]. Thus, although rehabilitation targeting proprioceptive input and reduced visual dependency is required, standardized protocols are lacking. Dysesthesia-matched transcutaneous electrical nerve stimulation (DM-TENS) has recently emerged as a novel therapeutic option for dysesthesia. DM-TENS applies electrical stimulation that matches the patient’s perceived dysesthesia (e.g., rhythm and intensity). This approach offers greater relief from dysesthesia compared to conventional high-frequency TENS and may enhance mechanical sensation [7]. However, despite the increasing interest in DM-TENS, to our knowledge, no study has specifically examined its impact on persistent lower-limb sensory disturbances or associated balance dysfunction following LSS surgery. In this case study, we aimed to investigate the effects of DM-TENS on persistent bilateral plantar dysesthesia and reduced ADL, QOL, gait, and balance after LSS. The effects of DM-TENS on dysesthesia and objective postural sway were assessed.

## Case presentation

A 70-year-old woman (height: 150 cm, weight: 50 kg, body mass index: 22.1 kg/m²) with L4/5 LSS presented with bilateral plantar dysesthesia that had gradually developed over the past year. In this report, the term “dysesthesia” refers specifically to the patient’s subjective complaints of numbness and discomfort in the plantar region, as rated using a numerical rating scale (NRS). Objective sensory abnormalities, such as allodynia or evoked pain, were not observed or assessed. One month before admission, she experienced worsening lower-extremity pain and new-onset urinary disturbances (retention requiring catheterization), prompting surgical admission. At the time of admission (postoperative day (POD) 2), she reported severe bilateral plantar numbness (NRS score: 8) and lower-extremity pain (NRS score: 7), which interfered with her sleep and daily activities. She expressed significant distress about her limited ability to move and perform household tasks. She underwent posterior lumbar interbody fusion and laminectomy at the L4/5 level. Her past medical history included angina pectoris. She had no history of diabetes mellitus or other systemic conditions suggestive of peripheral neuropathy. An indwelling urinary catheter was maintained during the early postoperative period. Tramadol hydrochloride (25 mg, three times daily) was prescribed for postoperative pain. The patient exhibited no signs of cognitive impairment. Premorbidly, she lived with her spouse, managed all household chores independently, and worked part-time (farming, milk delivery, and cleaning). Her primary rehabilitation goals were: “I want the numbness (dysesthesia) to go away, and I want to be able to do my housework as before.”

Standard PT and occupational therapy (OT) commenced on POD 2. The total duration and frequency of standard PT and OT sessions remained consistent between POD 2-14 and POD 14-30, with approximately 40 minutes of therapy provided once daily, five days per week. The initial assessment indicated a global lower extremity manual muscle testing (MMT) grade of 3/5. Surgical site pain was rated 7/10 on the NRS. Superficial plantar sensation was reduced (NRS score: 6/10, 10 = normal), and bilateral plantar dysesthesia was severe (NRS score: 8/10, 10 = worst). Sensory examination revealed that other modalities, pain, temperature, vibration, and position sense, were intact at baseline. Quantitative sensory testing was not conducted due to the lack of appropriate equipment in our clinical setting. Functional ambulation required a walker (functional ambulation category (FAC) level 2).

On POD 14 (pre-DM-TENS), the MMT grade had risen to 4/5 and surgical-site pain had fallen to NRS 1/10; however, plantar superficial sensation remained NRS 6/10 and plantar dysesthesia NRS 8/10. Psychological testing showed pronounced catastrophizing on the Pain Catastrophizing Scale (PCS; © 1995 Michael J. L. Sullivan, non-commercial licence-to-use granted by Mapi Research Trust, ID 117661, 22 Jun 2025) with a total score of 25 (rumination 14, helplessness 8, magnification 1) [8]. The Short-Form McGill Pain Questionnaire-2 (SFMPQ-2; numerical data only, permission not required per Wolters Kluwer) yielded a total of 40 responses (continuous: 15, intermittent: 13, neuropathic: 10, affective: 2) [9]. Although ambulation had improved to FAC 3, a walker was still required, and her ability to perform housework, a key personal goal, remained limited. Scores on the Canadian Occupational Performance Measure (COPM; permission granted via direct correspondence with COPM Inc.) were: importance 10, performance 3, and satisfaction 4 [10]. Despite gains in mobility and reduction of surgical pain, the patient expressed marked distress about the persistent dysesthesia: “Even though I had surgery, the numbness hasn’t improved at all. I can’t do housework or work like this when I go home.”

Considering the persistent nature of plantar dysesthesia and its substantial effects on the patient’s perceived function, ADLs, and psychological well-being, DM-TENS was introduced as an adjunct to the standard PT/OT program from POD 15, following discussions with the patient and rehabilitation team. A timeline detailing the key clinical events, interventions, and assessment points is provided in Supplementary Table S1. Detailed descriptions of the DM-TENS intervention and the standard PT/OT program following the Template for Intervention Description and Replication (TIDieR) checklist are presented in the Appendices.

Intervention procedure: standard rehabilitation and DM-TENS

Standard postoperative PT and OT were performed throughout the inpatient stay (Appendices). DM-TENS was applied adjunctively from POD 15 to 30. Additionally, the key parameters and procedures described in this section were employed. A low-frequency stimulator (ESPURGE®, Ito Co., Ltd., Tokyo, Japan) was used. Parameters (100 μs pulse width, 80 Hz) were selected to achieve synchronization with the patient’s perceived dysesthesia, following the methods described by Nishi et al. [7]. Two self-adhesive electrodes (5×5 cm) were placed on each plantar surface: one over the metatarsal heads and one over the calcaneus. Socks were used to secure the electrodes. DM-TENS was applied concurrently for 40 min per session during scheduled PT/OT sessions. The intervention was delivered twice daily (total 80 min/day), five days per week, for a total of 15 intervention days (30 applications). Each application began with the therapist verifying electrode contact and optimal stimulation-dysesthesia synchronization with the patient. Further details on the DM-TENS protocol are provided in the Appendices.

Analysis procedure (postural control assessment)

Center of pressure (COP) sway was measured using stabilometry (JK-101Ⅱ, UNIMEC Co., Ltd., Tokyo, Japan) on POD 15 and 30. The sampling frequency was 100 Hz. Measurements (30 s each) were performed under eyes-open (OE) and eyes-closed (CE) conditions (standing with the feet 20 cm apart). The tests were conducted in a quiet room, where the patient stood naturally and gazed forward.

Data were analyzed using R software (version 4.3.2; R Foundation for Statistical Computing, Vienna, Austria). Noise was reduced using a 10 Hz low-pass filter [11]. Raw COP data (X and Y) were mean-centered for visualization. The COP trajectory analysis included the 95% confidence ellipse area, total path length, mean velocity, and mean acceleration. The root mean square (RMS) displacement, mean position, velocity, and acceleration were calculated in the anteroposterior (Y) and mediolateral (X) directions. Time-domain COP plots were used to visualize time-series changes.

Power spectral density (PSD) analysis (Welch’s method [12]) was used to evaluate the frequency characteristics. The X and Y signals were decomposed into low (<0.3 Hz), mid (MF, 0.3-1 Hz), and high (HF, 1-3 Hz) frequency bands [12]. The PSD and mean power frequency (MPF) were calculated per band to assess the differences between OE and CE.

The PSD is typically presented as absolute power (e.g., cm²/Hz) or as a percentage of total power [13]. Here, each band’s PSD is expressed as a percentage of the total power (sum = 100%) to clarify the relative contributions. This normalization aids the comparisons across measurements/conditions.

Progress

At POD 30, the MMT grade improved (3→4) and surgical pain resolved (NRS score: 7→0). Superficial sensation improved (NRS score: 6→9) and plantar dysesthesia markedly decreased (NRS score: 8→3). The PCS scores improved notably (total: 25→16; rumination: 14→10; helplessness: 8→1). The SFMPQ-2 scores also showed substantial improvement (total: 40→13; continuous: 15→5; intermittent: 13→3; neuropathic: 10→5; affective: 2→0).

Functional ambulation improved (FAC 2→5), and the patient progressed from walking to using a cane. COPM scores for housework improved substantially (performance, 3→8; satisfaction, 4→8). The patient reported: “The numbness has gotten better, and it’s easier to move. I think I can manage housework without problems” (Table 1-3).

**Table 1:** Motor, sensory, and functional measures at baseline, pre-intervention, and post-intervention This table summarizes changes in lower extremity motor function, pain intensity, sensory status, and functional ambulation across the intervention period. Assessments were conducted on POD 2 (baseline), POD 14 (pre-intervention), and POD 30 (post-intervention). Improvements were observed in muscle strength, dysesthesia, and gait function, with the transition from walker to T-cane reflecting enhanced ambulatory capacity. MMT: manual muscle testing, NRS: numeric rating scale, FAC: functional ambulation categories, FIM: functional independence measure, POD: postoperative day

Assessment parameter	Baseline assessment (POD 2)	Pre-intervention assessment (POD 14)	Post-intervention assessment (POD 30)
Lower extremity MMT	3	4	4
Pain (NRS)	7	1	0
Plantar superficial sensation (NRS)	6	6	9
Plantar dysesthesia (NRS)	8	8	3
FAC	2	3	5
Ambulation aid	Walker	Walker	T-cane
FIM (motor score)	33	54	86

**Table 2:** Psychological distress and pain characteristics pre- and post-intervention This table displays pre-intervention (POD 14) and post-intervention (POD 30) scores on two patient-reported measures: Pain Catastrophizing Scale (PCS; © 1995 Michael J. L. Sullivan, non-commercial Licence-to-Use granted by Mapi Research Trust, ID 117661, 22 Jun 2025) [8], Short-Form McGill Pain Questionnaire-2 (SFMPQ-2; numerical data only, permission not required per Wolters Kluwer) [9]. Reductions in all subscales from POD 14 to POD 30 indicate a decrease in levels of catastrophizing and multidimensional pain following the DM-TENS intervention. Definitions of SFMPQ-2 subscales: continuous pain refers to ongoing pain sensations (e.g., aching, throbbing); intermittent pain involves pain that comes and goes (e.g., stabbing, shooting); neuropathic pain captures pain qualities linked to nerve dysfunction (e.g., tingling, burning); and affective descriptors reflect emotional responses to pain (e.g., tiring, fearful). PCS: Pain Catastrophizing Scale, SFMPQ-2: Short-Form McGill Pain Questionnaire-2, POD: postoperative day

Assessment parameter	Subscale	Pre-intervention assessment (POD 14)	Post-intervention assessment (POD 30)
PCS (score)	Total	25	16
	Rumination	14	10
	Helplessness	8	1
	Magnification	1	1
SFMPQ-2 (score)	Total	40	13
	Continuous pain	15	5
	Intermittent pain	13	3
	Neuropathic pain	10	5
	Affective descriptors	2	0

**Table 3:** Patient-reported outcomes related to housework and subjective perception across the intervention period This table outlines patient-reported outcomes related to housework performance, measured using the Canadian Occupational Performance Measure (COPM; permission granted via direct correspondence with COPM Inc.) [10], and corresponding subjective statements reflecting changes in perception across the intervention timeline. COPM scores for performance and satisfaction improved significantly after the intervention, and patient comments demonstrated increasing confidence and a reduction in distress. COPM: Canadian Occupational Performance Measure, POD: postoperative day

Assessment parameter	Subscale	Baseline assessment (POD 2)	Pre-intervention assessment (POD 14)	Post-intervention assessment (POD 30)
COPM - housework (score)	Importance	10	10	10
	Performance	-	3	8
	Satisfaction	-	4	8
Characteristic patient statements		Unable to move well due to surgical site pain	Even after surgery, the numbness hasn't improved at all. I'm unable to do anything like this	The numbness has improved, and I feel like I can move more easily. I think I can manage housework too

**Figure 1:**
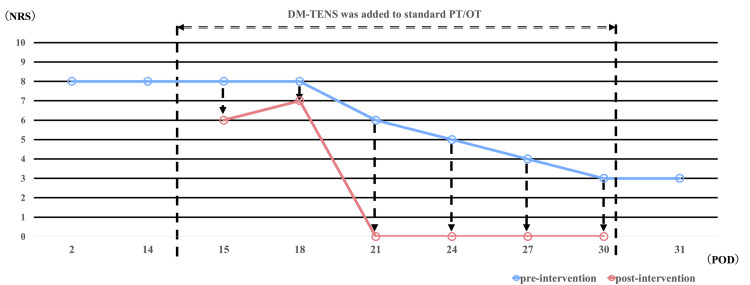
NRS scores for dysesthesia immediately before and after each DM-TENS session Blue bars represent pre-intervention scores, and red bars represent post-intervention scores. This figure displays the intensity of dysesthesia (NRS scores) immediately before and after each DM-TENS session. As this is a retrospective case report based on clinical records, daily measurements of dysesthesia were not consistently available. Data were documented approximately every three days, and only those time points with available records are presented. NRS: numerical rating scale, DM-TENS: dysesthesia-matched transcutaneous electrical nerve stimulation, PT: physical therapy, OT: occupational therapy, POD: postoperative day

Figure 1 illustrates the pre- and post-session NRS ratings for dysesthesia. Immediate post-session reductions occurred: POD 15 (8→6), POD 18 (8→7), POD 21 (6→0), POD 24 (5→0), POD 27 (4→0), and POD 30 (3→0). At discharge (POD 31), the dysesthesia remained low (NRS score: 4), suggesting sustained effects. From POD 21, the sessions resulted in complete and immediate resolution of the dysesthesia (NRS score: 0).

The pre-intervention (POD 15) and post-intervention (POD 30) stabilometry data are shown in Table 4. Postural sway improved under both the OE and CE conditions. Improvements were marked under CE: 95% confidence ellipse area (-24.2%, 109.23→82.81 cm²), total path length (-25.9%, 230.92→171.22 cm), and mean velocity (-25.8%, 7.70→5.71 cm/s). Anteroposterior (Y-axis) COP parameters also improved: RMS (-15.3%, 1.11→0.94 cm) and mean velocity (-25.2%, 6.98→5.22 cm/s).

The PSD analysis also showed changes, particularly in CE anteroposterior sway. Relative high-frequency power (HF, 1-3 Hz) decreased (36.93%→24.45%), whereas mid-frequency power (MF, 0.3-1 Hz) increased (46.94%→58.76%). The MPF decreased (1.01→0.83 Hz).

**Table 4:** Stabilometry parameters before (POD 14) and after (POD 30) DM-TENS intervention This table summarizes changes in COP parameters and PSD measures under eyes-open and eyes-closed conditions. Improvements were most notable under the eyes-closed condition, with reductions in sway area, velocity, and acceleration. Frequency-domain analysis showed a shift from high- to mid-frequency power, suggesting enhanced central postural control. Mean position change (%) and MPF change (%) were not calculated, as these values reflect positional shifts or are not interpretable via simple percentage change. ¹ LF power: <0.3 Hz, ² MF power: 0.3–1.0 Hz, and ³ HF power: 1–3 Hz COP: center of pressure, DM-TENS: dysesthesia-matched transcutaneous electrical nerve stimulation, PSD: power spectral density, RMS: root mean square, POD: postoperative day, ML: mediolateral, AP: anteroposterior, MPF: mean power frequency, OE: eyes open, CE: eyes closed, LF: low frequency, HF: high frequency

Parameter group	Parameter	OE condition			CE condition		
		Pre-intervention (POD 14)	Post-intervention (POD 30)	Change (%)	Pre-intervention (POD 14)	Post-intervention (POD 30)	Change (%)
Overall COP parameters	95% confidence ellipse area (cm²)	66	56.19	-14.87	109.24	82.82	-24.19
	Total path length (cm)	103.19	91.53	-11.3	230.92	171.22	-25.85
	Mean velocity (cm/s)	3.44	3.05	-11.3	7.7	5.71	-25.84
	Mean acceleration (cm/s²)	0.11	0.1	-11.33	0.26	0.19	-26.92
ML COP	RMS sway (cm)	0.5	0.44	-12.38	0.53	0.47	-11.32
	Mean position (cm)	0.54	1.24	-	1.1	1.6	-
	Mean velocity (cm/s)	1.28	0.89	-30.23	2.19	1.6	-26.94
	Mean acceleration (cm/s²)	0.04	0.03	-30.21	0.07	0.05	-28.57
AP COP	RMS sway (cm)	0.75	0.69	-8.43	1.11	0.95	-14.41
	Mean position (cm)	–0.0638	–4.6446	-	–0.0176	-4.5876	-
	Mean velocity (cm/s)	2.95	2.78	-5.89	6.98	5.22	-25.21
	Mean acceleration (cm/s²)	0.1	0.09	-5.9	0.23	0.17	-26.09
ML PSD	LF power (%)¹	61.44	85.53	24.1	63.08	60.23	-2.8
	MF power (%)²	26.01	12.26	-13.74	23.39	29.48	6.1
	HF power (%)³	12.56	2.21	-10.34	13.54	10.29	-3.252
	MPF (Hz)	0.46	0.25	-	0.51	0.43	-
AP PSD	LF power (%)¹	44.1	67.71	23.6	16.14	16.79	0.6
	MF power (%)²	37.76	23.37	-14.39	46.94	58.76	11.8
	HF power (%)³	18.14	8.92	-9.22	36.93	24.45	-12.48
	MPF (Hz)	0.62	0.42	-	1.01	0.83	-

**Figure 2:**
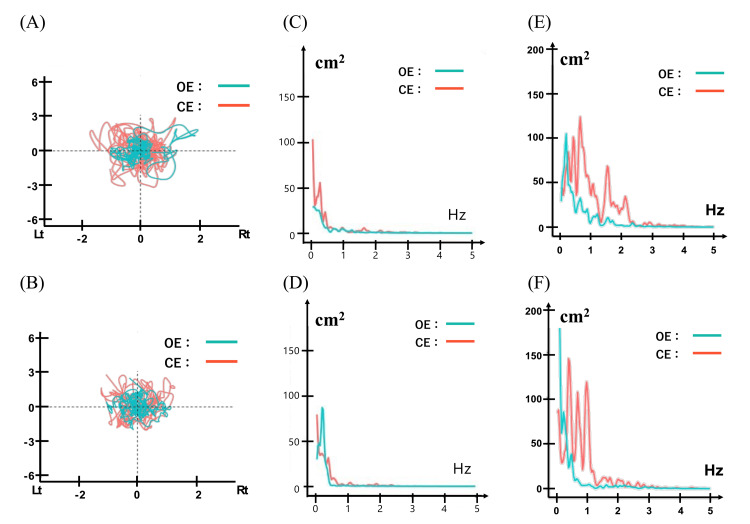
Postural sway characteristics pre- and post-intervention with DM-TENS on POD 14 and POD 30 OE (teal lines) and CE (orange lines) conditions (A) Pre-intervention COP trajectory. (B) Post-intervention COP trajectory, showing a reduction in sway area. (C) Pre-intervention ML sway velocity PSD. (D) Post-intervention ML sway velocity PSD. (E) Pre-intervention AP sway velocity PSD. (F) Post-intervention AP sway velocity PSD, demonstrating a shift toward lower and mid-frequency bands, particularly under CE conditions. COP: center of pressure, ML: mediolateral, AP: anteroposterior, PSD: power spectral density, OE: eyes open, CE: eyes closed, POD: postoperative day, DM-TENS: dysesthesia-matched transcutaneous electrical nerve stimulation

The COP trajectory plots (Figure 2A-2B) showed a reduced sway area at POD 30. The PSD analysis (Figure 2C-2F) indicated a power shift toward the low-to-mid frequency range (0.3-1 Hz) post-intervention. The patient completed all scheduled DM-TENS sessions with good tolerability and no adverse events.

Ethical considerations

Written informed consent for participation and publication (including images) was obtained from the patient. This single-patient case report was exempt from ethics review under the policy of Central Japan International Medical Center. The report was prepared in accordance with the CARE guidelines [14]; the completed CARE checklist is provided in the Appendices.

## Discussion

In this case report, we demonstrate that DM-TENS can alleviate persistent plantar dysesthesia after LSS surgery and enhance objective postural control. DM-TENS was associated with a marked reduction in dysesthesia intensity, improvement in plantar sensation, and clear gains in stabilometric indices (total path length, elliptical area, and mean velocity). These changes extend earlier work, indicating that DM-TENS outperforms conventional high-frequency TENS for sensory relief [7].

Pre-intervention, the patient exhibited abnormally large CE anteroposterior sway, typical of patients with LSS whose proprioceptive input is degraded by peripheral neuropathy [7,15]. After the two-week DM-TENS program, the sway magnitude and velocity approached the published normative ranges, suggesting a genuine sensory-driven improvement rather than merely a natural postoperative recovery.

The power spectral analysis indicated a shift from high-frequency (>1 Hz) to mid-frequency (0.3-1 Hz) dominance under the CE condition. High-frequency power is generally attributed to ankle-level reflex-driven adjustments, whereas the mid-frequency components reflect more centrally integrated cerebellar-vestibular control [16,17]. Therefore, the observed shift implies a transition from effortful reactive ankle strategies to a more efficient, centrally coordinated balance control. This pattern aligns with the sensory reweighting concept, in which enhanced plantar afferent precision enables the nervous system to downweight unreliable visual information and upweight somatosensory cues [18,19].

Psychological and functional outcomes followed a similar trajectory. Reductions in catastrophizing coincided with improved ambulation (FAC) and higher self-rated performance and satisfaction (COPM). This supports a cascading pathway of improved sensation, refined postural control, enhanced motor function, and improved ADL and QOL, which is consistent with pain neuroscience models of chronic spinal conditions [20].

Therefore, this case provides preliminary evidence that sensory improvements induced by DM-TENS may translate into functional balance gains in individuals with persistent postsurgical neuropathic foot symptoms. This condition is notoriously refractory to conventional treatments. Clinically, DM-TENS may offer a simple, noninvasive adjunct to conventional rehabilitation programs for addressing postoperative dysesthesia and fall risk in older adults.

Nevertheless, this case report has several limitations. First, as a single-case report, generalizability is limited, and treatment effects may vary depending on individual patient characteristics (e.g., age, sex, stenosis severity, surgical procedure). Second, although improvements were observed during the postoperative period, distinguishing DM-TENS-specific effects from natural recovery remains challenging. However, previous studies have indicated that postoperative dysesthesia may persist for years in a substantial proportion of LSS patients [5], suggesting that spontaneous resolution alone may not explain the observed changes. Third, no neurophysiological assessments (e.g., nerve conduction studies) were conducted, leaving the possibility of subclinical neuropathy unresolved. Fourth, although stimulation parameters were adjusted based on the patient's dysesthesia characteristics, they were not systematically optimized, and no established standard for parameter individualization currently exists [6]. Finally, the long-term effects beyond POD 31 remain unknown, and the durability of symptom relief cannot be evaluated.

Given these limitations, future studies should adopt rigorous designs, such as single-case experimental designs, crossover trials, or multicenter randomized controlled trials, to verify the efficacy, safety, and long-term effects of DM-TENS in diverse populations. Research should also aim to identify predictors of treatment response, explore neurophysiological mechanisms using objective assessments, and develop standardized protocols for parameter optimization. Integrating DM-TENS into comprehensive rehabilitation programs, including exercise and psychological interventions, may enhance recovery in patients with persistent neuropathic sensory disturbances after LSS surgery.

Patient perspective

"Before the surgery, my main hope was that the terrible numbness and burning feeling in the soles of my feet would finally disappear. So, when weeks passed after the operation and it felt the same, maybe even worse some days, I felt really disappointed and quite worried. I started thinking I'd never be able to walk properly again or return to my normal life, including my chores and jobs. When my therapist explained this new type of electrical stimulation (DM-TENS) that was supposed to match my numbness, I was honestly skeptical, having tried other things before without much luck. Still, I was willing to try anything at that point. The first time they put it on, it was a strange sensation, a specific kind of tingling that, surprisingly, seemed to almost "tune in" or resonate with the rhythm of my numbness. It wasn't unpleasant. Day by day, during the 40-minute sessions, I began to notice that the background numbness felt a little less intense afterwards. It wasn't a sudden miracle, but gradually, over the two weeks, my feet started to feel clearer, less heavy, less 'wrong'. Walking became noticeably easier, and I felt less afraid of losing my balance. Now, the numbness is much better - not completely gone, but significantly more manageable. I can feel the floor under my feet properly again, and I'm back doing my housework without constantly focusing on the discomfort. It gave me my hope back."

## Conclusions

This case report demonstrates the potential therapeutic benefit of DM-TENS in alleviating persistent plantar dysesthesia and improving postural control following LSS surgery. The intervention led to reductions in dysesthesia intensity and improvements in proprioception, psychological state, and objective balance performance, especially under eyes-closed conditions. These findings highlight the importance of targeting sensory dysfunction in post-LSS rehabilitation and suggest that DM-TENS may serve as a simple, noninvasive adjunct to standard therapy. Further studies using rigorous designs are warranted to confirm its efficacy, mechanisms, and long-term effects.

## Figures and Tables

**Figure 3:**
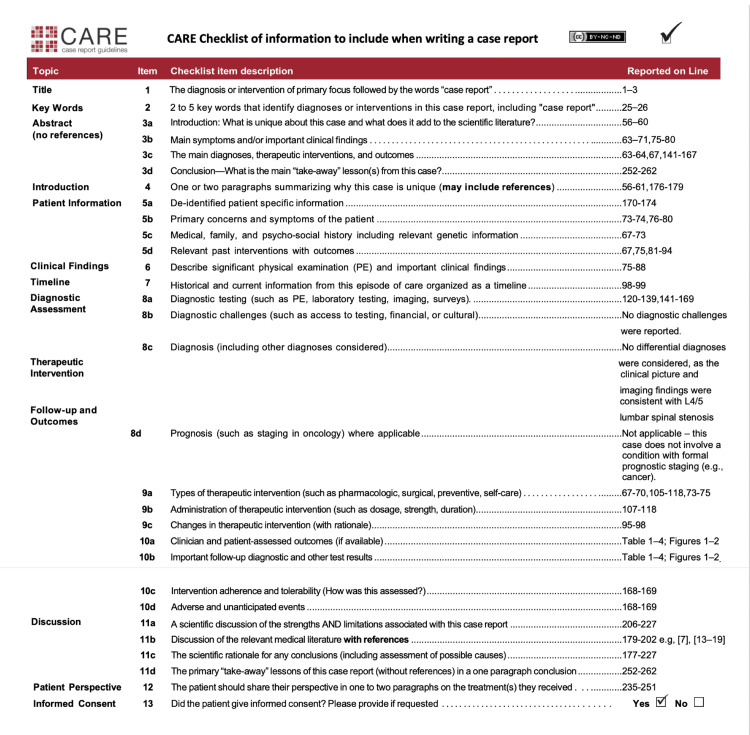
CARE checklist for case report compliance This checklist was completed in accordance with the CARE guidelines to ensure transparency and completeness in reporting this case. The line numbers correspond to the locations in the main manuscript where each checklist item is addressed. Reproduced from Gagnier et al., 2014 [14], with permission from Elsevier. CARE: case report

## Appendices

**Table 5:** Timeline of key clinical events and interventions “→” indicates continuation of a symptom or intervention, “⇨” indicates timing of DM-TENS sessions, “+” indicates presence of symptom, “−” indicates resolution of symptom, “↓” indicates symptom improvement, and “■” indicates assessment time point. Baseline was defined as the initial state before any intervention. Pre-intervention and post-intervention refer to time points immediately before and after the DM-TENS phase, respectively. PT: physical therapy, OT: occupational therapy, DM-TENS: dysesthesia-matched transcutaneous electrical nerve stimulation, POD: postoperative day

Event/intervention	~1 y prior	...	-1 month	...	Admission (POD -1 approx.)	Surgery (POD 0)	POD 2	...	POD 14	POD 15	...	POD 30	POD 31 (discharge)
Standard rehabilitation (PT/OT)							→	→	→	→	→	→	
DM-TENS intervention										⇨	⇨	⇨	
Assessments point							■		■			■	■
							Baseline		Pre-intervention			Post-intervention	Discharge
Bilateral plantar dysesthesia	+	→	→	→	→	→	→	→	→	→	↓	↓	→
Lower limb pain/urinary disturbance			＋	→	→	→	→	→	-				

**Table 6:** Intervention details based on the TIDieR checklist Adapted from Hoffmann et al., 2014. BMJ. 2014;348:g1687. doi:10.1136/bmj.g1687 TIDieR: template for intervention description and replication, PT: physical therapy, OT: occupational therapy, DM-TENS: dysesthesia-matched transcutaneous electrical nerve stimulation, POD: postoperative day, NRS: numeric rating scale, OE: eyes open, CE: eyes closed, ADL: activity of daily living

TIDieR item	Detail	PT/OT	DM-TENS
1. Name	Brief name of the intervention	Standard postoperative rehabilitation (PT + OT)	DM-TENS
2. Why	Rationale or theory for the intervention	Improve muscle strength, balance, gait, and ADL to facilitate functional recovery after lumbar surgery	Electrical stimulation synchronized with perceived dysesthesia aims to normalize aberrant plantar sensory processing, potentially via gate control or central modulation mechanisms (Nishi et al., 2022 [7])
3. What: materials	Physical or informational materials used	Basic rehabilitation equipment (e.g., exercise mats, weights (up to 0.5 kg)), walking aids (standard walker, T-cane)	ESPURGE® low-frequency electrical stimulator (Ito Co., Ltd., Tokyo, Japan), two 5 × 5 cm self-adhesive electrodes per foot, hypoallergenic tape or socks for securing electrodes
4. What: procedures	Key elements, actions, and processes	Exercises included: supine straight-leg raises, standing heel raises; static and dynamic weight-shifting exercises, single-leg stance practice (OE/CE), tandem stance balance; over-ground gait training (progressing from walker to T-cane); task-oriented ADL training simulating housework activities	Parameters: biphasic rectangular pulses, 100 µs pulse width, 80 Hz frequency. Intensity: adjusted to a strong but comfortable sensory level perceived by the patient as matching the quality/rhythm of their intrinsic dysesthesia. Electrode placement: one over the metatarsal heads, one over the calcaneus, bilaterally. Session workflow: verify electrode contact → adjust intensity for optimal subjective synchronization → stimulate for the prescribed duration. Synchronization was confirmed at the start of each session
5. Who provided	Expertise, background, specific training	Licensed physiotherapist with 10 years of clinical experience in orthopedic rehabilitation	Same physiotherapist delivered the intervention
6. How	Mode of delivery	One-to-one, face-to-face sessions	Applied directly by the therapist concurrently during scheduled PT/OT sessions
7. Where	Location(s) of intervention delivery	Inpatient rehabilitation gym at the hospital	Same location
8. When and how much	Dosage, frequency, session length, duration	40 min per session, once daily, five days per week, initiated on POD 2 and continued until discharge (approx. 20 sessions planned over the relevant period)	40 min per application, twice daily (total 80 min per day), five days per week, from POD 15 to POD 30 (total of 15 sessions/30 applications planned)
9. Tailoring	Planned individualization or adaptation	Exercise intensity/difficulty progressed based on patient tolerance and performance (e.g., adding 0.5 kg of resistance if surgical site pain was rated NRS ≤2/10). Gait aid progressed from walker to T-cane based on stability and therapist assessment	Stimulation intensity was adjusted at each session based on patient feedback to ensure optimal synchronization with the perceived dysesthesia, aiming for a maximal comfortable sensation without causing pain
10. Modifications	Changes to the protocol during the study	No significant modifications were made to the standard PT/OT protocol during the observation period pertinent to this case report	No modifications were made to the core DM-TENS protocol (parameters, duration, frequency) during the intervention period. Intensity adjustments were part of the planned tailoring
11. How well: fidelity	Methods to monitor/improve delivery quality	The designated physiotherapist supervised all sessions. Patient safety was monitored via heart rate, blood pressure, and pain NRS assessment (session paused if pain NRS ≥4/10)	The designated physiotherapist supervised all applications. Synchronization was rechecked at the start of every session. Skin integrity under the electrodes was inspected before and after each application
12. How well: adherence	Methods to monitor/improve patient adherence	Patient attendance at scheduled sessions was recorded	Patient attendance and completion of DM-TENS applications were recorded
Concomitant Care	Other relevant care	Oral tramadol hydrochloride 25 mg three times daily was prescribed for pain management; dosage remained unchanged throughout the PT/OT and DM-TENS intervention periods	Received standard PT/OT sessions concurrently as described in the adjacent column. No other new modalities or pain medications were introduced during the DM-TENS intervention period

## References

[1] Ishida K, Azuma Y, Tsushima E (2024). The effect of combining muscle strengthening and aerobic exercise on patients with lumbar spinal canal stenosis (Article in Japanese). Phys Ther Jpn.

[2] Konno S, Kikuchi S, Tanaka Y (2007). A diagnostic support tool for lumbar spinal stenosis: a self-administered, self-reported history questionnaire. BMC Musculoskelet Disord.

[3] Watanabe K, Sekiguchi M, Yonemoto K (2017). Bowel/bladder dysfunction and numbness in the sole of the both feet in lumbar spinal stenosis - a multicenter cross-sectional study. J Orthop Sci.

[4] Ruan Y, Cheng J, Dai J (2023). Chronic stress hinders sensory axon regeneration via impairing mitochondrial cristae and OXPHOS. Sci Adv.

[5] Katz JN, Zimmerman ZE, Mass H, Makhni MC (2022). Diagnosis and management of lumbar spinal stenosis: a review. JAMA.

[6] Sasaki K, Senda M, Katayama Y, Ota H, Matsuyama Y (2013). Characteristics of postural sway during quiet standing before and after the occurrence of neurogenic intermittent claudication in female patients with degenerative lumbar spinal canal stenosis. J Phys Ther Sci.

[7] Nishi Y, Ikuno K, Minamikawa Y, Igawa Y, Osumi M, Morioka S (2022). A novel form of transcutaneous electrical nerve stimulation for the reduction of dysesthesias caused by spinal nerve dysfunction: a case series. Front Hum Neurosci.

[8] Sullivan MJL, Bishop SR, Pivik J (1995). The pain catastrophizing scale: development and validation. Psychol Assess.

[9] Dworkin RH, Turk DC, Revicki DA (2009). Development and initial validation of an expanded and revised version of the Short-form McGill Pain Questionnaire (SF-MPQ-2). Pain.

[10] Law M, Baptiste S, McColl M, Opzoomer A, Polatajko H, Pollock N (1990). The Canadian occupational performance measure: an outcome measure for occupational therapy. Can J Occup Ther.

[11] Scoppa F, Capra R, Gallamini M, Shiffer R (2013). Clinical stabilometry standardization: basic definitions--acquisition interval--sampling frequency. Gait Posture.

[12] Vieira MF, de Avelar IS, Silva MS, Soares V, Lobo da Costa PH (2015). Effects of four days hiking on postural control. PLoS One.

[13] Fujimoto C, Kamogashira T, Kinoshita M (2014). Power spectral analysis of postural sway during foam posturography in patients with peripheral vestibular dysfunction. Otol Neurotol.

[14] Gagnier JJ, Kienle G, Altman DG, Moher D, Sox H, Riley D (2013). The CARE guidelines: consensus-based clinical case reporting guideline development. BMJ Case Rep.

[15] Kneis S, Bruetsch V, Dalin D, Hubbe U, Maurer C (2019). Altered postural timing and abnormally low use of proprioception in lumbar spinal stenosis pre- and post- surgical decompression. BMC Musculoskelet Disord.

[16] Lin IS, Lai DM, Ding JJ (2019). Reweighting of the sensory inputs for postural control in patients with cervical spondylotic myelopathy after surgery. J Neuroeng Rehabil.

[17] St-Amant G, Rahman T, Polskaia N, Fraser S, Lajoie Y (2020). Unveilling the cerebral and sensory contributions to automatic postural control during dual-task standing. Hum Mov Sci.

[18] Peterka RJ (2002). Sensorimotor integration in human postural control. J Neurophysiol.

[19] Maurer C, Mergner T, Peterka RJ (2006). Multisensory control of human upright stance. Exp Brain Res.

[20] Neblett R, Hartzell MM, Williams M, Bevers KR, Mayer TG, Gatchel RJ (2017). Use of the Central Sensitization Inventory (CSI) as a treatment outcome measure for patients with chronic spinal pain disorder in a functional restoration program. Spine J.

